# Retraction: Molecular detection of eukaryotes in a single human stool sample from Senegal

**DOI:** 10.1371/journal.pone.0346619

**Published:** 2026-04-07

**Authors:** The PLOS One Editors

The *PLOS One* Editors retract this article [1,2] due to concerns about compliance with the PLOS Human Subjects Research policy.

The Abstract and the Materials and Methods section in [1] report that the study involved stool samples obtained from a volunteer in Senegal. The ethics approval statement reported in this article states that both this study and the assent procedure were approved by the National Ethics Committee of Senegal (CNERS) and the Ethics Committee of the Institut Fédératif de Recherche IFR 48, Faculty of Medecine, Marseille, France (agreement number N° 09–022). PLOS notes that agreement number N° 09–022 was also reported in at least 247 other studies despite apparent differences in the aims and objectives, study locations, study populations, age ranges, methodologies, types of samples collected, and types of consent described in these studies. S1 File contains a summary of articles citing ethics approval number N° 09–022 of which PLOS is aware.

A representative of the Aix-Marseille Université Ethics Committee stated that the institutional investigation into the ethics concerns concluded this article meets ethical standards. Furthermore, the representative indicated that they were not concerned about the ethics approval number reuse and stated that N° 09–022 is a “generic” approval applying to any situation where the process is unchanged. They provided the ethics approval documents N° 09–022 and N° 00.86 MSP/DS/CNERS for editorial review.

PLOS reviewed the documentation provided by the institution and concluded that the documents did not fully resolve the journal’s concerns. Specifically,

- Ethics approval document N° 09–022 issued on 09 December 2009 by the Comité d’Ethique de l’IRF48 for a study titled “*Culture diversifiée des bactéries dans les selles humaines*”. It allows for the use of clinical stool samples originating from the bacteriology laboratory of Timone Hospital, Marseille, France. The ethics approval N° 09–022 does not specify an approved study period, nor does it specify approval for sample collection from volunteers abroad.
- Ethics approval document N° 00.86 MSP/DS/CNERS issued on 02 June 2010 by the Ministère de la Santé et de la Prévention of the République de Sénégal for a study titled “*Identification des agents pathogènes responsibles de fièvre au Sénégal. Réalisation de tests diagnostiques chez les malades consultant dans les dispensaires de Dielmo et Ndiop*”*.* It grants approval for carrying out diagnostic tests on patients in the Dielmo and Ndiop dispensaries with the aim of identification of pathogens responsible for fever in Senegal. The study described in the document does not appear to match the study reported in [1], and the document does not mention approval for the secondary analysis of the collected samples as part of a separate study.
- PLOS identified potential competing interests between the committee that granted ethics approval N° 09–022 and one or more of the article’s authors.

In addition to the ethics approval concerns listed above, the *PLOS One* article [1] did not report sufficient information about participant recruitment and eligibility criteria as would be needed to replicate this study, and the findings of the study reported in this article [1] appear to be based on a stool sample obtained from a single individual, raising concerns about the reliability and reproducibility of the results reported in this study. Furthermore, this article [1] did not report when the sample used in this study was collected. This information is needed to evaluate the article’s compliance with the PLOS Human Subjects Research policy.

CS did not agree with the retraction. IH, DR, and FB either did not respond directly or could not be reached.

## Supporting information

S1 File Overview of 248 articles referencing ethics approval number N°09 – 022. (XLSX)

## References

[1] Hamad I, Sokhna C, Raoult D, Bittar F. RETRACTED: Molecular detection of eukaryotes in a single human stool sample from Senegal. PLoS One. 2012;7(7):e40888. doi: 10.1371/journal.pone.0040888 22808282 PMC3396631

[2] The *PLOS ONE* Editors. Expression of concern: Molecular detection of eukaryotes in a single human stool sample from Senegal. PLoS One. 2022;17(12):e0277999. doi: 10.1371/journal.pone.0277999 36512567 PMC9747042

